# Forcing a square into a circle: why South Africa’s draft revised material transfer agreement is not fit for purpose

**DOI:** 10.3389/fphar.2024.1333672

**Published:** 2024-03-12

**Authors:** Paul Esselaar, Lee Swales, Devarasi Bellengère, Banele Mhlongo, Donrich Thaldar

**Affiliations:** School of Law, University of KwaZulu-Natal, Durban, South Africa

**Keywords:** data, decolonial, human biological material, material transfer agreement, ownership, pathogens, POPIA, stewardship

## Abstract

The South African National Health Research Ethics Council (NHREC) recently released a final draft revision of the standard material transfer agreement (MTA) that was promulgated into law in 2018. This new draft MTA raises pertinent questions about the NHREC’s mandate, the way in which the draft MTA deals with data and with human biological material, and its avoidance of the concept of ownership. After South Africa’s data protection legislation, the Protection of Personal Information Act (POPIA), became operational in mid 2021, the legal landscape changed and it is doubtful that the NHREC has a residual mandate to govern personal information in health research. Furthermore, data is dealt with in a superficial, throw-away fashion in the draft MTA. The position with human biological material is not substantially better, as the draft MTA fails to recognise that human biological material can contain pathogens, which has important legal and ethical ramifications that are not sufficiently addressed. A central problem with the draft MTA is its use of the term ‘steward’, and avoidance of the legal concept of ‘ownership’. This is not only misaligned with the South African legal framework, but also fails to consider the ethical case for recognising ownership. Finally, a call to embrace decolonial thinking in health research underscores the importance of recognising ownership in order to foster the growth of the local bio-economy. Key recommendations to reshape the draft MTA include: Making use of the eventual revised MTA optional, and allowing it to evolve with input from scientific and legal communities; regulating the transfer of associated data in a separate data transfer agreement that can be incorporated by reference in the MTA; enhancing guidance on liability and risk management in respect of human biological material that contains pathogens; and, finally, adopting a decolonial approach in health research governance, which requires recognising the ownership rights of South African research institutions.

## Background

In 2018, the South African Minister of Health published a standard material transfer agreement (MTA) in the Government Gazette and gave notice that research institutions sharing human biological material for health research or clinical trials must use this MTA (SA MTA) (Material Transfer Agreement for Human Biological Materials, 2018). The SA MTA was controversial from the outset (Thaldar, 2020; Thaldar et al., 2020). The notion of the state in a supposedly open and democratic society, forcing the use of a single template onto everyone is clearly suspect. The situation could, however, have been palatable had the SA MTA been a well-drafted document. However, it was not. Thaldar et al. (2020) highlighted several problems with the SA MTA, ranging from misalignment with extant law to absurdly overbroad clauses.

The only saving grace was that the SA MTA described itself as a “framework,” hence leaving latitude for parties that are legally forced to use it to amend the substantive provisions—and hopefully in the process resolve the problematic aspects (Thaldar et al., 2020; Steytler and Thaldar, 2021; Thaldar and Shozi, 2021; Swales et al., 2023a). Using this latitude, a group of South African law academics developed a revised version of the SA MTA in an attempt to rectify the most serious issues while remaining within the bounds of the framework of the original version of the SA MTA (Pope, 2020). The aim of this revised version—called “SA MTA 1.1” and dating from 2020—was to provide the South African research community with a *usable* version of the SA MTA that would still comply with the law.

Next, in 2022, a research group at the University of KwaZulu-Natal in South Africa started with the development of a data transfer agreement (DTA) template for the South African research community. The rationale was that data sharing between researchers requires an expertly drafted agreement that is aligned with South African law—in particular the Protection of Personal Information Act 4 of 2013 (POPIA) that was brought into full operation on 1 July 2021; however, many—if not most—research organisations in South Africa do not have the inhouse legal expertise to have such an agreement drafted (Swales et al., 2023a). Accordingly, the aim was to develop a comprehensive, professionally drafted DTA template and to make it freely available for anyone to use (Swales et al., 2023a). The DTA template was also complemented with an explanatory memorandum to guide users on how to use and amend the template for their own circumstances (Swales et al., 2023b).

The authors of the DTA template explicitly distanced themselves from the authoritarian practice of forcing the use of a document on a country (Swales et al., 2023a; Swales et al., 2023b). Instead, they stated that the South African research community should use the DTA template and explanatory memorandum because these are top quality documents that answer a need, not because they are forced to do so as is the case with the SA MTA (Swales et al., 2023a; Swales et al., 2023b).

However, the controversial SA MTA (the original version) remained in South Africa’s lawbooks. Eventually, South Africa’s Department of Health decided that the best policy solution was to task a statutory body that functions under its aegis, the National Health Research Ethics Council (NHREC), with revising the SA MTA. In August 2023, the NHREC distributed a final draft version of their revised SA MTA to stakeholders for comment (National Health Research Ethics Council, 2023). An interesting observation is that the NHREC’s final draft is largely based on SA MTA 1.1, rather than on the original SA MTA. Thus, the NHREC’s final draft benefits from avoiding the well-documented pitfalls of the original SA MTA. However, the NHREC made some consequential changes to SA MTA 1.1. It is also important to note that the South African legal landscape has changed since SA MTA 1.1 was developed. We have already mentioned POPIA’s coming into operation. This raises the important question of whether the NHREC’s final draft MTA is aligned with POPIA?

In the sections that follow, we delve into a comprehensive examination of the NHREC’s draft MTA and its implications for South Africa’s research community and their international collaborators. We investigate four questions: First, do the Minister of Health and the NHREC have the mandate to regulate data in the health research context, or are they overstepping their respective mandates? Second, does the draft MTA provide sufficient protection for data? Third, is there sufficient guidance on biological material in the draft MTA? Fourth, why does the draft MTA shy away from the concept of ownership? Flowing from our analyses of these four questions, we propose an alternative approach to the draft MTA, and offer recommendations to address the identified shortcomings and to align the draft MTA with legal standards and with the needs of the scientific community.

## Main text

### Are the Minister of Health and the NHREC overstepping their respective mandates?

At a fundamental level, the question must be posed: Do the Minister of Health and the NHREC have the mandate to regulate data in the health research context, or are they overstepping their respective mandates? These entities receive their regulatory mandates from the National Health Act 61 of 2003 (NHA). Chapter 8 of the NHA, in particular, together with relevant regulations, governs the use of human biological material and research with human participants. However, there is also a later statute that is relevant in the health research space, namely POPIA, which deals with personal information. Data in the health research space often includes personal data—or to use POPIA terminology, “personal information.” Moreover, data in the health research space are often *sensitive personal* data—or to use POPIA terminology, “special personal information.” Accordingly, there is an overlap between the scopes of application of the NHA and POPIA. The question then is: In the case of a conflict, which statute prevails? We consider two relevant legal principles.

In the context of health research, the NHA is *general* legislation, while POPIA is *special* legislation, meaning that POPIA governs only a specific part of health research, namely the way in which the personal information of research subjects is dealt with (National Health Act, 2003). Accordingly, the maxim *generalia specialibus non derogant* (general words and rules do not derogate from special ones) applies. This means that the scope of application of the general statute must be constrained by the presence of the specific legislation (Minister of Justice and Constitutional Development v Southern African Litigation Centre, 2016). Applied to health research, this principle means that the governance of personal information is now governed by POPIA first and the NHA second. It follows that the Minister of Health and the NHREC—who get their respective mandates from the NHA—no longer have a mandate to regulate personal information in the health research milieu. This is now done by POPIA and its implementation mechanism, the Information Regulator. In turn, the Information Regulator can, among others, issue guidance notes, and approve codes of conduct and compliance frameworks. For example, ASSAf developed a draft Code of Conduct for Research (Academy of Science of South Africa, 2023), which is likely to be converted into a compliance framework based on the Information Regulator’s feedback.

Furthermore, POPIA itself contains a supremacy clause in section 2(a). In the context of the processing of personal information, POPIA (2013) supersedes any other legislation that is inconsistent with it. There is however an exception to POPIA’s supremacy clause in section 2(b). If any other legislation provides for conditions for the lawful processing of personal information that are “more extensive” than those set out in POPIA, the more extensive conditions in the other legislation prevail. Although some have argued that the NHA is more extensive (in the sense that it is certainly more voluminous), this is mistaken (Bronstein and Nyachowe, 2023). In context, “more extensive” clearly refers to *better protection of data subjects*, not to being more voluminous (Thaldar, 2023). This exception may apply in specific instances where other legislation provides better protection of data subjects. However, as we discuss below, this is evidently not the case with the draft MTA. Accordingly, there is no realistic possibility of relying on the exception to POPIA’s supremacy clause.

As a result, to the extent that the NHREC’s draft MTA contains provisions regarding personal information, it is beyond the Minister of Health’s and the NHREC’s statutory mandate. The Minister of Health and the NHREC are overstepping into the terrain of the Information Regulator. To the extent that they overstep, their conduct is invalid and can be challenged in a court of law (Sasol Oil Pty Ltd v Metcalfe, 2004). The solution to this problem is obvious: The NHREC should remove all references to “associated data” in its draft MTA.

Next, we analyse the way in which the draft MTA deals with “associated data.”

### Is there sufficient protection for the associated data in the draft MTA?

Although trite, it bears repetition: POPIA sets out eight conditions for the lawful processing of personal information (De Stadler et al., 2021; Burns and Burger-Smidt, 2023). These conditions are aimed at protecting the rights of data subjects, but POPIA also recognises that a balance must be struck between the right to privacy and the right of access to information and freedom of speech. POPIA therefore establishes conditions that regulate how personal information may be processed. For the avoidance of doubt, POPIA applies to the processing (including transfer) of all personal information, including personal information derived directly and indirectly from health research, such as genetic data generated from human biological material.

In terms of current best practice—in South Africa and internationally—an agreement that facilitates the transfer of data containing personal information should contain *detailed provisions* articulating compliance with applicable data protection legislation. Parties to an MTA must be aware that by transferring data that contains personal information, several legal obligations arise—and these obligations require careful consideration. The parties must determine, *inter alia*, the nature of the personal information being transferred, the identity of the responsible party, and the data privacy obligations on each party. Critically, sections 107 and 109 of POPIA (2013) provides that failure to comply with POPIA can result in a fine of up to R10 million or imprisonment for a period not exceeding 10 years, or to both a fine and such imprisonment—as well as significant reputational harm.

However, the draft MTA fails to live up to best practice. The draft MTA’s “Guidance” section notes that the draft MTA is a template that contains “minimum standards.” However, as it stands, there are simply no minimum standards in the draft MTA dealing with data protection. The draft MTA refers to data in a superficial, throw-away fashion.

The “Guidance” section further provides that where “data alone” is transferred a data transfer agreement (DTA) is “appropriate.” We suggest that in all circumstances where data containing personal information are transferred, in order to ensure full compliance with POPIA, and to abide by international best practice, a DTA is not only appropriate, but necessary. Although it is true that some of the content in a DTA will be similar to a MTA, the similarity relates only to standard legal clauses, and not to the actual substance of the agreement. The primary purpose of the agreements will be entirely different, and both will seek to comply with distinct pieces of legislation. For this reason, the decision to conflate data with human biological material—something inherited from the original SA MTA via SA MTA 1.1—is a mistake.

To illustrate the issues caused by this conflation, consider the following three definitions:

• “Material” is defined as including both human biological material and associated data.
• “Associated data” includes personal information relating to human biological material.
• “Permit” is defined as “authorisation of the National Department of Health to transfer and/or export Material.”

However, in relation to personal information (which is part of ‘Material’ as defined above), the National Department of Health plays no role in its regulation.

Some of the changes that the NHREC’s draft MTA introduced to SA MTA 1.1 seem not to have been sufficiently considered. For example, consider the second sentence added to clause 3.5. The clause now reads as follows: “The Provider must inform the HREC [health research ethics committee] and wherever possible the Participant/s if the Provider is informed that the Material has Become Identifiable for any reason whatsoever. This must be clarified as Material remain [sic] coded and hence potentially identifiable.” The second sentence is not comprehensible.

Another example is the definition of “Becomes Identifiable.” In the draft MTA the word “directly” was added before “personally identified.” This is ill-advised, as it makes the draft MTA narrower than POPIA, which can lead to inconsistency and confusion.

The NHREC’s draft MTA is inadequate in relation to the transfer of data. We suggest that the conflation between data and biological material be avoided. These concepts should be dealt with distinctly, as they are governed by different disciplines in the law. Preferably, the envisioned MTA should avoid regulating the transfer of data altogether—rather, it should only regulate the transfer of human biological materials to avoid misalignment with POPIA. In conjunction with such a pure MTA, parties must consider the use of a professionally drafted DTA that takes account of applicable legislation and is designed to lawfully manage the processing of data. Here there is a ready solution, namely the DTA template that was developed for South Africa’s research community. It is fully aligned with POPIA and freely available (Swales et al., 2023b).

Next, we move the focus from the incorporeal to the corporeal—from data to biological material.

### Is there sufficient guidance on biological material in the draft MTA?

It is interesting that the NHREC’s draft MTA—similar to its predecessors—focuses only on human biological material, to the exclusion of other biological material that is important in health research, such as human pathogens. However, ‘Human Biological Material’ is defined sufficiently broadly in the draft MTA as to include pathogens. The definition reads as follows:

‘Human Biological Material’ means a biological sample or tissue from a person, living or deceased, including Deoxyribonucleic Acid (DNA), Ribonucleic Acid (RNA), blastomeres, polar bodies, cultured cells, embryos, gametes, progenitor stem cells, growth factors and blood specimens, biopsy tissue and any modifications or derivatives thereof

Consider the following scenario: When, during a pandemic, blood samples are drawn from infected persons and sent from one research institution to another, the blood sample would qualify as “Human Biological Material.” However, such “Human Biological Material” would also contain a human pathogen, such as a bacteria or a virus. This is a matter of concern, as the draft MTA does not sufficiently cater for such a possibility. For a researcher there is a vast difference between a human biological material sample that contains a pathogen and a sample that does not, and the procedure for dealing with each is quite different.

Ultimately, the person that the revised SA MTA will govern will be the person who will need to organise the transfer of human biological material—which may include pathogens—and so they need to be aware of the legal and physical dangers relating to this. It may not be apparent that human biological material could be a weapon of mass destruction and yet that is exactly what it could be if the human biological material contains certain pathogens, such as the Ebola virus. This is acknowledged in the Non-Proliferation of Weapons of Mass Destruction Act (1993) and yet the draft MTA does not mention this important consideration. International standards, such as the WHO Manual on Laboratory Biosafety (World Health Organisation, 2020), the National Institutes of Health Shipping Policies and Procedures (National Institutes of Health, 2022), the International Air Transport Association’s Infectious Substances Shipping Regulations (International Air Transport Association, 2023), and the Centers for Disease Control and Prevention’s Guideline for Disinfection and Sterilization in Healthcare Facilities (Centers for Disease Control and Prevention, 2008) are examples of useful links. However, these are also omitted from the draft MTA leaving it up to scientists to source the relevant material on their own. In this respect, the draft MTA misses a vital opportunity to help and educate scientists by alerting them to the requirements that they need to comply with in order to transfer certain kinds of human biological material.

Apart from missing this opportunity to create awareness among scientists, the issue of pathogens being present in human biological material also opens up the issue of legal liability. At present, the draft MTA includes a provision that obliges the recipient to indemnify the provider of material from any liability, except insofar as the provider is required to be liable in law. The recipient is also required to maintain “adequate” insurance cover against liability to third parties. However, the draft MTA provides no assistance as to when the provider will be liable in terms of the law, nor does it require checks and balances to avert the harm that may or may not be covered by the “adequate” insurance. It is important to consider that the agreement may deal with the transfer of a biological weapon of mass destruction and so liability could be huge, possibly even worldwide. It is unlikely that this type of harm could be cured by any insurance cover and therefore greater effort should be invested in the eventual revised SA MTA to ensure that the harm does not occur.

The provider of the biological material should consider the infectious nature, volume and frequency of the transfer (among other factors) when considering the risk posed by the transfer of the human biological material. The identified risks would also influence the safeguards the provider would need to adopt. In this regard, the process to identify and deal with risks as set out in section 19(2) of POPIA could be considered to be a template for this purpose. The provider can, for example, create an appropriate risk matrix to be added as an annexure to the agreement. In addition, a right to audit compliance by either party should be included. The exercise of this right should be based on the risk profile of the other party.

We now proceed to the last research question, which pertains both to human biological material and associated data: the issue of ownership.

### Why shy away from ownership?

#### The legal ownership of human biological material

The NHA is clear that the only way in which a research participant can provide a sample of his or her bodily material for research, such as tissue or blood, is by *donating* it to a research institution (section 63). Donation is a legal technical term for a nominate contract that entails the *transfer of ownership* from the donor to the donee (Mankowitz v Loewenthal, 1982, para. 765A; Thaldar and Shozi, 2021). Accordingly, when a research participant provides a sample of his or her bodily material for research, the *only legal way* in which this can transpire is for the research participant to *transfer ownership* to the research institution (DE v CE, 2020, para. 24; Thaldar and Shozi, 2021). That means that the research institution is the *owner* of the human biological material that it collects for research (Thaldar and Shozi, 2021).

Moreover, the Regulations regarding the General Control of Human Bodies, Tissue, Blood, Blood Products and Gametes (2012) also provides that a person who acquires human biological material in terms of the NHA acquires *exclusive rights* in such human biological material (Regulation 26). This is not only consistent with the transfer of ownership to the research institution, but it also makes it clear that the transfer of ownership must be absolute and unqualified (National Health Act, 2003, s. 63; Regulations regarding the General Control of Human Bodies, Tissue, Blood, Blood Products and Gametes, 2012, reg. 24). In other words, the donor is not allowed to retain any rights whatsoever in the donated human biological material.

However, despite these clear statutory provisions, the NHREC decided to obfuscate and confuse the issue by introducing the concept of a “steward”—a concept that is not part of any branch of South African law that is relevant to health research (National Health Research Ethics Council, 2023). The NHREC defines “steward” as “a person or entity *entrusted* by the Participant to *safeguard and protect* the Material” (National Health Research Ethics Council, 2023) (Emphasis added). This is misaligned with ownership, for two reasons: First, an owner has the *right to destroy* the owned object—most certainly *not the duty* to “safeguard and protect” the owned object (Pope, et al., 2020). Second, the word “entrusted” points to a trust relationship between the research institution and the research participant with respect to the donated material (National Health Research Ethics Council, 2023). This is in conflict with the Regulations regarding the General Control of Human Bodies, Tissue, Blood, Blood Products and Gametes (2012) which provides that the research institution enjoys *exclusive* rights in the donated material (Regulation 26).

South Africa’s NHA was enacted by the democratically elected representatives of the people of South Africa. It embraces ownership of human biological material by research (National Health Act, 2003, s. 63; Regulations regarding the General Control of Human Bodies, Tissue, Blood, Blood Products and Gametes, 2012, reg. 24). However, the NHREC is not respecting the democratic process. The NHREC is promoting ownership-denial. We suggest that the NHREC should take the law of South Africa more seriously.

#### The legal ownership of data

“Material” as defined in the MTA includes “associated data.” The problematic nature of conflating these two very different kinds of object—human biological material and data—into one term was highlighted above. “Associated data” is defined as “the information associated with the Human Biological Material, including personal information, derived directly or indirectly prior and during the conduct of the research Project” (National Health Research Ethics Council, 2023). Accordingly, associated data includes all data—personal and non-personal—that are in any undefined way ‘associated’ with the human biological material (National Health Research Ethics Council, 2023). Superficially, the notion of a *data* steward seems to make sense, given that POPIA (2013) places various duties on a responsible party in relation to personal data. These statutory rights of the data subject qualify the common law ownership rights that a research institution may have in the personal data (Protection of Personal Information, 2013). However, the problem that lurks below the surface is that associated data as defined in the MTA are not limited to personal data but can also include de-identified data (Thaldar, et al., 2020). Consider that POPIA (2013) applies only to *personal* data, and ceases to apply when that same data is not personal or is de-identified to become non-personal data (section 3(1)). However, the NHREC’s final draft would have a data steward safeguard and protect associated data even if it is not personal data. This makes no sense and is counter-productive. Moreover, the creation of a data steward does not consider the role of the Information Officer, who plays a crucial role in POPIA.

Using human genomic sequence data as an example, and applying the well-established requirement for private ownership in South African law, Thaldar et al. (2022) argue that a data instance—i.e., the computer file containing the data—is a digital object that is susceptible of private ownership in South African law. The authors further consider the rules concerning the acquisition of ownership in South African law, and suggest that the research institution that generates genomic sequence data is in the best position to acquire ownership in the data instances that it generates (Thaldar et al., 2022). In line with this conclusion, the DTA template embraces data ownership (Swales et al., 2023a; Swales et al., 2023b). Because data is a new kind of object and data ownership is not yet well established in the law, it is *essential* that data owners—South African research institutions—should clearly and explicitly record their ownership of the data that is being shared in their DTAs (Swales et al., 2023a).

Some may think that since data is incorporeal, ownership of data is an *intellectual* property right. However, this is mistaken. As analysed by Thaldar et al. (2022), common law ownership is not limited to corporeal objects. In fact, at least since the Second Century, when the Roman jurist Gaius wrote his Institutes, property law included incorporeal objects (Gaius, 1946). More recent examples of private ownership of incorporeal objects are, *inter alia*, digital money, digital books, and digital music (Nightingale v Devisme, 1770; Nissan South Africa Pty Ltd v Marnitz, 2006; S v Ndebele, 2012; Competition Commission v British American Tobacco South Africa Pty Ltd, 2009; S v De Vries, 2008; Curemed CC v Van Onselen, 2015). Millions of people buy music (as digital objects) on their smart phones using digital money (which is also a digital object). *Intellectual* property law, by contrast, is a more recent branch of the law, mostly found in statute and not in common law, and only applicable to specifically defined *kinds* of incorporeal objects, such as inventions and artistic creations (Copyright Act, 1978, s. 2; Patents Act, 1978, s. 3). It is however possible for intellectual property rights to overlap with common law property rights (Thaldar et al., 2022). Intellectual property law would typically not apply directly to data, but rather indirectly (Thaldar et al., 2022). This would be the case if, for example, data is used in an invention (patent law) or as part of a database (copyright law) (Copyright Act, 1978; Patents Act, 1978). However, the application of intellectual property law in no way overrides or supplants ownership in a data instance (Thaldar et al., 2022). Various rights can co-exist and qualify one another. For example, if one buys a book, one becomes the owner of the book, but the author still retains copyright in the content (Thaldar et al., 2022). The author’s copyright qualifies the owner’s rights in the sense that the book owner may not make copies of the book without the author’s consent (Thaldar et al., 2022). The ways in which the rights emanating in various branches of South African law interact in the context of data are explored in detail by Thaldar et al. (2022).

#### The ethical case for owning the data that one generates

Not only is there a solid *legal* case for data ownership in the health research context, but there is also an *ethical* case, provided by John Locke’s labour theory of property (Locke, 1963). In brief, this entails that persons ought to acquire ownership in the fruits of their own labour (Locke, 1963). Applied to the generation of data, it is the research institution that *collects* the pheno-clinical data from research participants, and that *generates* genetic and genomic data by sequencing DNA isolated from samples donated by research participants. In other words, the research institution is the party that invests its *labour* into producing the data, and therefore ought to own such data. In health research, this typically requires significant investment in expensive equipment and highly trained human resources. Accordingly, it is ethically justified for the research institution to actively claim the fruits of its labour. Why does the NHREC shy away from supporting research institutions to claim what they are ethically entitled to?

#### Decolonial thinking about health research

In the colonial way of thinking about health research, global health research is conceptualised as an eternal cycle where Africa provides raw “genetic resources” to the Global North, while the Global North conducts value-added research on the “genetic resources” of Africa and owns the intellectual property in inventions such as new precision medicines, which are then sold to Africa for profit. Although this colonial way of thinking about health research is based on historical and (sometimes at least) current facts, it can become self-perpetuating when simply assumed and used as the basis for policy-making.

Allow us to explain: If policymakers make it more difficult for commercial research companies to acquire and control human biological samples and derivatives therefrom, such as DNA, cell-lines and data, the policymakers may think—because of the colonial paradigm in which they conceive health research—that they are protecting Africa from possible exploitation. However, what they may also be doing at the same time is to suppress the growth of the nascent biotechnology sector in Africa itself. In this way, the policy measure that are intended to protect Africa have the perverse effect of ossifying the colonial power structure and hence perpetuating the colonial paradigm of conceiving health research.

We therefore call for *decolonial thinking about health research*. Policymakers should reflect on their paradigms and how their resulting policy decisions can self-perpetuate the colonial power structures. Policymakers should actively strive to think anew about health research, and envision a (future) vibrant and sustainable African bio-economy, and then consider what policy choices would best assist the country to achieve that vision. To the extent that the NHREC has decided to become involved in health research policy development—revising the SA MTA is indeed policy development—the NHREC members should ensure that they are intimately familiar with South Africa’s *Bio-economy Strategy* (Department of Science and Technology, 2013). If their answer is “but our mandate is ethics,” then they should rethink why they have taken up the project of revising the SA MTA.

Firmly acknowledging research institutions’ *data ownership* is not only ethical, but also core to developing a bio-economy that can compete globally in the Knowledge Economy. In the decolonised vision that we propose, South African biotech companies will act in lawful and ethically appropriate ways towards research participants, including respecting the research participants’ privacy rights in the personal data that relate to them. In this way, the data owner can also have a “custodian” or “steward” function, by ensuring the safety of personal data. However, without clarity on ownership, being a mere “custodian” or “steward” is legally toothless (Thaldar, 2024). Furthermore, in the decolonised vision that we propose, South African biotech companies will build South Africa’s bio-economy by generating a wealth of data. These data can be used for research in South Africa, and can be monetised by licencing access to such data in trusted research environments, or, where such data is de-identified, licencing access in less restrictive ways, such as data transfers. However, if a biotech company is merely the “steward” of the data that it generates, with uncertainty about ownership, there is *no legal basis* for any of these commercial actions (Thaldar, 2024). *Ownership* provides this essential legal basis (Thaldar, 2024). Without it, South Africa will be a *knowledge colony*.

## Conclusion

At this point, it must be clear that we believe that the NHREC’s draft revised version of the SA MTA is misdirected in several respects and the entire paradigm underlying the creation and content of the draft MTA needs to be considered anew. The NHREC needs to return to first principles to determine what they seek to achieve with a revised SA MTA and whether those are appropriate goals. In order to assist with this, we have the following four main recommendations on how the draft MTA could be reimagined:

### Recommendation 1: make the use of the SA MTA voluntary, not mandatory

The draft MTA is not a mature document and it will take some time for it to reach a level of maturity where it is appropriate for it to be considered to be mandatory. The scientific and legal community should be encouraged to work together to progressively improve on the content of the draft MTA and to stress-test it against the actual lived experience of scientists who transfer human biological material. In addition, a mandatory document is inherently less flexible—and thus less able to be updated regularly—than a voluntary document.

### Recommendation 2: data should be dealt with separately

The transfer of human biological material and the transfer of data are different disciplines, and different legal rules apply. Moreover, the current definition of “Associated Data” in the draft MTA merges the concept of personal and non-personal information in an unfortunate and unhelpful manner which contributes to confusion. If there are any associated data transferred alongside human biological material, it would be more appropriate to indicate that the DTA template developed for the South African research community (Swales et al., 2023b) as amended by the parties would govern such data.

### Recommendation 3: enhanced liability and risk management provisions

Given that the draft MTA involves handling human biological materials—which can contain pathogens—the potential for harm is significant. The MTA should require providers of human biological material to assess the transfer risk by considering factors such as the infectious nature, volume, and frequency of the transfer. Recognising these risks will determine the necessary safeguards that can also be built into the eventual revised SA MTA, such as contractual warranties by the provider. Moreover, the eventual revised SA MTA should include a provision allowing either party to audit compliance, with the decision and scope of the audit informed by the other party’s risk profile.

### Recommendation 4: adopt a decolonial approach in the governance of health research

In the context of South Africa, it is crucial to ensure that health research does not perpetuate colonial legacies. Adopting a decolonial approach entails having a clear vision of a thriving bio-economy in South Africa—built not merely on being a raw material provider, but on adding value to such material—and strategically aiming for policy decisions that achieve this vision. Clarity on ownership of human biological material and associated data (primary and inferential) is crucial in order to have confidence and certainty in transactions entailing the transfer of these (corporeal and incorporeal) objects. This in turn is vital for building a thriving bio-economy in South Africa. Accordingly, policy instruments such as the eventual revised SA MTA should strive to empower local research institutions by clearly recognising their legal ownership of the material that they share.

Note that the draft MTA was not made public by the NHREC. Instead, the NHREC disseminated it to the health research ethics committees, who were given the opportunity to submit comments.
